# Association between direct government subsidies and service scope of primary care facilities: a cross-sectional study in China

**DOI:** 10.1186/s12939-020-01248-7

**Published:** 2020-08-10

**Authors:** Zhong Li, Peiyin Hung, Ruibo He, Liang Zhang

**Affiliations:** 1 School of Medicine and Health Management, Tongji Medical College, Huazhong University of Science and Technology, Wuhan, 430030 Hubei China; 2 Research Center for Rural Health Services, Key Research Institute of Humanities & Social Sciences of Hubei Provincial Department of Education, Wuhan, 430030 Hubei China; 3 Arnold School of Public Health, University of South Carolina, Columbia, 29205 SC USA; 4 School of Finance and Public Administration, Hubei University of Economics, Wuhan, 430205 Hubei China

**Keywords:** Direct government subsidies, Financial revenue, Service scope, Primary care facility, China

## Abstract

**Background:**

Comprehensive primary care practices, through preconception, preventive, curative, and rehabilitative care, have been a global priority in the promotion of health. However, the scope of primary care services has still been in decline in China. Studies on the factors for primary care service scope have centred on human resources and infrastructure; the role of direct government subsidies (DGS) on services scope of primary care facilities were left unanswered. This study aimed to explore the association between the DGS and services scope of primary care facilities in China.

**Methods:**

A multi-stage, clustered cross-sectional survey using self-administrated questionnaire was conducted among primary care facilities of 36 districts/counties in China. A total of 770 primary care facilities were surveyed with 757 (98.3%) valid respondents. Of the 757 primary care facilities, 469 (62.0%) provided us detailed information of financial revenue and DGS from 2009 to 2016. Therefore, 469 primary care facilities from 31 counties/districts were included in this study. Sasabuchi-Lind-Mehlum tests and multivariate regression models were used to examine the inverted U-shaped relationship between the DGS and service scope.

**Results:**

Of 469 PCFs, 332 (70.8%) were township health centres. Proportion of annul DGS to FR arose from 26.5% in 2009 to 50.5% in 2016. At the low proportion of DGS to financial revenue, an increase in DGS was associated with an increased service scope of primary care facilities, whereas the proportion of DGS to financial revenue over 42.5% might cause narrowed service scope (*P* = 0.023, 95% CI 11.59–51.74%); for the basic medical care dimension, the cut point is 42.6%. However, association between DGS and service scope of public health by primary care facilities is statistically insignificant.

**Conclusion:**

While the DGS successfully achieved equalization of basic preventive and public health services, the disproportionate proportion of DGS to financial revenue is associated with narrowed service scope, which might cause underutilization of primary care and distorted incentive structure of primary care. Future improvements of DGS should focus on the incentive of broader basic medical services provision, such as clarifying service scope of primary care facilities and strategic procurement with a performance-based subsidies system to determine resource allocation.

## Background

Worldwide, health systems face financial pressure from increasing utilization of hospital-based services and expenditures, which are often unplanned, undesirable and avoidable [1–3]. As a fundamental element of the healthcare delivery system, primary care facilities (PCFs) play a vital role in the care coordination and the transition to specialized care [2, 4]. Many countries have made substantial efforts to improve their scope of care, thus improving the continuity of care and the performance of the primary care system. A comprehensive scope of care has been proven to be associated with reductions in medical expenditures (− 1.7%), hospitalizations (− 2.4%), and emergency department (ED) visits (− 2.5%) between the population in the highest and lowest quartiles of comprehensiveness of care [5]. In China, PCFs are often managed by township-level or community-level government [6]. With the substantial amount of government subsidies in infrastructure construction, workforce training and salaries since the Healthcare Reform in 2009, the government had gradually started to cover preventive and public health services, infrastructure and a large proportion of salaries. Healthcare services outside of traditional face-to-face office visits were also gradually paid or reimbursed, such as long-term care and chronic care management [7, 8].

Despite notable progress in the workforce, infrastructure and government subsidies, a large gap between effective care delivery and the needs of individuals and communities exists [9]. The maldistribution of human resources between primary and specialty care also hindered the service scope of PCFs [10]. Studies have revealed that the service scope of PCFs is declining despite their potential benefits [11, 12], which might exacerbate current geographic disparities in healthcare services availability and utilization [13]. Moreover, comprehensiveness of care received less resources and attention than other elements of primary care, such as access or continuity of care [14, 15].

Even though a performance-based salary (PBS) system was introduced to incentivize primary care providers, it was ineffective and did not fully encourage PCFs to provide diverse healthcare services [16]. First, PCFs generally did not link too many quality indicators with the PBS system, which might be a disincentive for healthcare providers to deliver more quality care [17]. A large proportion of PCFs are closing their surgical services [6], obstetrics and gynaecology services and other services [18]. Second, although the current payment system has set a higher reimbursement ratio for primary care services, primary healthcare providers are not adequately paid to provide services [17]. The fee-for-service payment system made comprehensive care less lucrative than highly profitable outpatient or inpatient services. The service scope of PCFs is narrowing under the rapid expansion of hospitals [16, 19]. Third, the hospital-centric healthcare delivery system is still expanding. The share of primary care outpatient visits to the total outpatient visits both from PCFs and hospitals decreased from 66% in 2009 to 57% in 2017 [17]. PCFs and hospitals do not provide services based on their designated function, which causes the healthcare system to be fragmented and inefficient [17].

To improve the service scope of primary care, the Chinese government has paid more attention to the service scope of PCFs with a continuous programme of capacity-building of PCFs [20]. Broader services have been proposed, such as Traditional Chinese Medicine (TCM), rehabilitation, hospice care, and home care. However, no definitive evidence on its service scope has been determined, although abundant studies revealed that training, patients’ or physicians’ preference, inappropriate insurance reimbursement, and salary incentives were associated with the decreased utilization of primary care [6, 18, 21]. One previous study indicated that primary care providers’ incentives will be distorted if financial support from government cannot be guaranteed [22]. Distorted incentive structures have made the Chinese health care system inefficient [17, 22]. PCFs should be encouraged and reimbursed to provide more services [23]. One report by the World Health Organization also revealed that efficient management of government input is vital to achieve universal health coverage [24]. Moreover, financial viability is essential to secure the service volume of primary care providers. Financial autonomy is associated with the achievement of desired goals and outcomes of primary care systems [25].

However, the role of direct government subsidies (DGS) on the scope of primary care services remains unclear. To fill the evidence gap, we aimed to investigate the association between the proportion of DGS to financial revenue (FR) and the service scope of the PCFs, thus facilitating early detection of the narrowed scope of primary care services and informing the capacity-building policies for PCFs at the risk of poor performance. The proportion of DGS to FR among rural and urban PCFs increased from 23% and 25% in 2010 to 37% and 45% in 2017, respectively. The proportion of healthcare services provided by the PCFs decreased by 7% from 2005 to 2015 [16]. This problem indicated that too much DGS might not lead to sufficient provision and utilization of primary care services. Therefore, we hypothesized that there is a threshold for the association between the proportion of DGS to FR and the service scope of PCFs. In other words, as the proportion of DGS to FR increases, the service scope of PCFs will substantially increase. However, once the proportion increases to a certain degree, the service scope will narrow.

## Methods

### Study design and data collection

A national, multistage, retrospective clustered survey of PCFs was conducted. First, six provinces/municipalities were randomly selected from 34 provincial regions according to geographic location and level of economic development (Appendix 1 in Table 4). Second, 10 prefectures were selected from the above 5 provinces per same principles (except Chongqing), given that the number of counties (rural areas) is twice that of districts (urban areas) in China. Two counties were randomly selected with the sample principles in all prefectures, and one district was randomly selected in each prefecture (8*2 + 8*1 = 24). In Chongqing, a municipality directly under the Central Government, four counties and two districts were directly selected (4 + 2 = 6). In Guangdong, as Shenzhen is highly urbanized, two districts were selected in Shenzhen, and four counties were randomly selected in Shaoguan (4 + 2 = 6). All PCFs in the 36 counties/districts were surveyed (Appendix 2 in Table 5) [26]. The self-reported service scope in 2017 was collected with a web-based survey under the coordination of the chief or deputy chief of each PCF. Administrative officers from the local department of health and health insurance in the study sites were also interviewed to collect facility-level and county-level characteristics from 2009 to 2016. Facility-level characteristics, including FR and DGS and human resources, were retrieved from the National Direct Online Reporting System. Finally, of 770 PCFs sampled, 757 (98.3%) valid responses were obtained. Of the 757 PCFs, 469 (62.0%) provided detailed information on FR and DGS from 2009 to 2016. Therefore, 469 PCFs from 31 counties/districts were included in this study.

### Outcome variable

Many definitions of the “scope of primary care” have been proposed by different studies, as well as the core scope of primary care practice [27–31]. Bazemore et al. created a scale to measure comprehensiveness by assessing 12 practices (i.e., emergency care, urgent care, major surgery, maternity care, office surgery, pain management, palliative care, postoperative care, preoperative care, prenatal care, newborn care and obstetrical deliveries); the score of the scale ranged from 0 to 12 [30]. Coutinho et al. measured the intended scope of practice of family medicine residents with by assessing clinical activities on a scale with scores ranging from 0 to 32 [31].

Based on previous studies [30, 31], the outcome of this study was the facility-level scope of primary care services. It was divided into two categories: preventive and public health services and basic medical care services. Preventive and public health services were combined with 12 items as the National Basic Public Service Specifications in 2017: 1) residents’ health records, 2) health education, 3) vaccination, 4) health management of children aged 0–6, 5) maternal health care, 6) health management of elderly people, 7) chronic disease management, 8) health management of patients with severe mental disorders, 9) health management of tuberculosis patients, 10) health management by TCM, 11) reporting of and response to infectious disease and public health emergencies, and 12) health inspection and supervision [20]. Basic medical care services were categorized into 20 items according to the guidelines of capacity-building for primary care facilities in China: 1) internal medicine, 2) surgical care, 3) paediatrics services, 4) gynaecology services, 5) obstetrics services, 6) dental care, 7) referee services, 8) home care, 9) telemedicine services, 10) general practice services, 11) family practice services, 12) TCM, 13) rehabilitation services, 14) mental health services, 15) ED services, 16) hospice care, 17) basic anaesthesiology for minor procedures, 18) medical laboratory services, 19) medical imaging services, and 20) electrocardiography services [20]. The services scope score was calculated according to cumulative service items provided for each facility and ranged from 1 to 32, with higher numbers representing broader scope of services.

### Independent variable

Proportion of cumulative DGS to FR (PCDGS) from 2009 to 2016.

### Control variables

As shown in Table 1, we included facility- and county-level characteristics for control variables per framework documented by one previous study [32].

**Table 1:** Variable explanation

Variable	Explanation
Service Scope	Scope of primary care services provided by primary care facilities in 2017
PCDGS (%)	Proportion of cumulative direct government subsidies to financial revenue of each facility from 2009 to 2016
PercapitaCFR	Per capita cumulative financial revenue of each facility from 2009 to 2016 (Chinese Yuan)
PercapitaCDGS	Per capita cumulative direct government subsidies to each facility from 2009 to 2016 (Chinese Yuan)
PercapitaGDP	Per capita gross domestic product at the county-level in 2017 (Chinese Yuan)
Pubhospitalave	Average number of public hospitals per 100,000 population in 2017
Privchospitalave	Average number of private hospitals in 100,000 population in 2017
Res	Number of facility-level residents in 2017, representing the potential need
Type	Type of primary care facilities: 1 = township health centre (THC), 2 = community health centre (CHC)
IDS	Whether primary care facility is integrated with high-level hospitals in 2017: 1 = Yes, 2 = No
MedStaff	Average density of registered physician and nurse in each thousand in 2017
MedStaffHighlevel	Average density of registered physician and nurse with high technical titles in each thousand in 2017
PBS	Proportion of performance-based bonus to the salary package in 2017. It often ranged from 30 to 40%

#### Facility-level characteristics

1) per capita cumulative FR and DGS from 2009 to 2016 (Chinese Yuan); 2) the number of township-level or community-level residents was used to represent the potential health need; 3) urban/rural: PCFs were categorized into township healthcare centres (THCs) and community healthcare centres (CHCs); 4) the status of the integrated delivery system in 2017 was used to represent the potential collaboration with delivery network [33]; 5) the number of medical staff and medical staff with high-level technical titles per thousand population was used to represent workforce; 6) the proportion of medical staff with high-level technical titles [6]; 7) the proportion of PBS to total salary package was used to represent internal incentive to medical staff [17].

#### County-level characteristics

1) per capita gross domestic product collected from the county-level census data was used to describe county-level economic development [6]; 2) the average number of public hospitals and private hospitals per 100,000 population were used to describe the intensity of competition [32].

### Statistical analysis

First, DGS and FR from 2009 to 2015 were adjusted to the current price in 2016 based on the annual consumer price index. The normality of the distribution of service scope scores was tested to determine which model should be used. Poisson regression model was used to examine the association between PCDGS and the service scope of PCFs (Shapiro-Wilk test of normality of preventive and public health score: *P* <  0.001). Second, independent variable and control variables were compared between PCFs categorized into four groups based on the quartiles of the service scope score. Third, because the limited higher-level sample size (a sample of 50 or less) could lead to biased estimates of the second-level standard errors for the two-level regression model [32, 33], we used the ordinary least squares (OLS) regression model to examine the association between the PCDGS and the service scope of PCFs. Multicollinearity was assessed with the variance inflation factor (VIF > 10). In this step, we first added all the included independent and control variables into the OLS regression model, and based on the estimates of the variance inflation factor, we excluded the variable assessing per capita cumulative DGS from 2009 to 2016 (VIF = 13.0). To reduce the bias of omitted variables, we then performed Ramsey’s regression equation specification error test [34]. The results (F = 2.88, *P* = 0.038) indicated that we should add the quadratic term of PCDGS into our regression model regardless of whether it would generally lead to multicollinearity between the PCDGS and quadratic term of PCDGS (VIF > 10) [35]. Fourth, the hypothesis of an inverted U-shaped relationship was tested by the approach proposed by Lind and Mehlum [36]. All statistical procedures were conducted with Stata 14.0. The significance level was set as α = 0.05.

## Results

### Basic characteristics

As shown in Table 2, of the 469 PCFs, 332 (70.8%) were THCs. A total of 362 (77.2%) PCFs were enrolled into integrated delivery systems in 2017. The PCDGS was 48.2% among the 469 PCFs. The proportion of annual DGS to FR increased from 26.5% in 2009 to 50.5% in 2016. PCFs in the second quantile of service scope scores reported the highest proportion of DGS to FR from 2009 to 2016, except for 2010. This result indicated that the association between the PCDGS and service scope might be nonlinear. The differences in the per capita cumulative FR (*P* <  0.001), per capita cumulative DGS (*P* <  0.001), per capita gross domestic product (*P* <  0.001), average counts of public hospitals (*P* <  0.001) and private hospitals (*P* = 0.02) per 100 thousand population, residents (*P* <  0.001), type (*P* <  0.001), medical staff per one thousand population (*P* <  0.001), and medical staff with high-level technical titles (*P* = 0.004) among the four quantiles were statistically significant. In addition, the differences in being enrolled in the integrated delivery system (*P* = 0.62), the proportion of high-level medical staff (*P* = 0.16), and the proportion of performance-based salary (*P* = 0.12) among the quantiles were not statistically significant.

**Table 2:** Facility characteristics by service scope of primacy care facilities in 2017

Variable	Overall ***N*** = 469	Quantile (1) ***N*** = 130	Quantile (2) ***N*** = 125	Quantile (3) ***N*** = 98	Quantile (4) ***N*** = 116	***P***
PCDGS (%)	47.3 (35.4, 58.9)	42.8 (34.0, 57.9)	52.2 (37.0, 63.1)	48.8 (33.9, 57.4)	47.3 (39.7, 54.9)	0.09
Proportion (%) 2016	50.5 (17.6)	50.1 (19.6)	53.1 (18.9)	51.1 (16.4)	47.8 (14.4)	0.13
Proportion (%) 2015	47.5 (35.4, 58.9)	43.1 (33.2, 57.9)	52.6 (37.0, 63.1)	49.0 (34.0, 57.4)	47.6 (40.1, 54.9)	0.08
Proportion (%) 2014	43.3 (34.3, 54)	41.2 (34.0, 53.5)	46.4 (36.5, 60.3)	43.1 (33.2, 54.5)	43 (34.3, 50.9)	0.07
Proportion (%) 2013	44.2 (34.7, 53.8)	44.8 (37.8, 52.8)	48.4 (39.0, 56.8)	43.6 (30.9, 53.0)	39.5 (32.0, 50.5)	**< 0.001**
Proportion (%) 2012	43.7 (35.0, 55.6)	44.5 (35.8, 56.0)	48.0 (37.2, 58.5)	43.2 (32.9, 55.1)	40.6 (33.9, 49.9)	**0.003**
Proportion (%) 2011	44.8 (35.5, 56.6)	44.2 (34.3, 56.0)	49.3 (40.5, 61.3)	44.4 (35.0, 54.8)	43.7 (32.7, 53.2)	**0.012**
Proportion (%) 2010	31.2 (23.0, 43.5)	33.7 (24.6, 44.3)	32.0 (23.1, 44.6)	30.4 (20.4, 42.3)	29.9 (22.3, 41.7)	0.38
Proportion (%) 2009	26.5 (15.0, 38.8)	29.6 (18.2, 43.7)	32.1 (17.7, 41.0)	24.4 (14.9, 35.0)	19.9 (12.5, 34.5)	**< 0.001**
PercapitaCFR	28.1 (19.8, 41.5)	22.6 (16.4, 33.7)	32.8 (19.7, 47.1)	26.6 (19.8, 37.5)	31.4 (24.8, 48.0)	**< 0.001**
PercapitaCDGS	13.3 (8.4, 21.4)	10.4 (7.1, 16.2)	15.7 (8.4, 27)	13.8 (7.9, 18.7)	15.2 (10.9, 21.4)	**< 0.001**
PercapitaGDP	5.9 (3.4, 11.4)	11.4 (3.7, 11.4)	7.8 (3.3, 11.4)	3.6 (3.2, 9.8)	3.7 (3.4, 8.9)	**< 0.001**
Pubhospitalave	0.6 (0.4, 1.7)	0.5 (0.3, 0.8)	0.6 (0.3, 1.6)	0.6 (0.5, 2.0)	0.8 (0.6, 2.8)	**< 0.001**
Prihospitalave	1.3 (0.6, 2.3)	0.9 (0.4, 2.3)	1.6 (0.4, 2.4)	1.4 (0.9, 2.3)	1.4 (0.9, 2.3)	**0.02**
Res	21.6 (12.3, 36.9)	18.9 (10.3, 30.6)	16.4 (9.3, 27.4)	24.4 (16.8, 37.9)	30.0 (18.7, 47.9)	**< 0.001**
Type						
THC	332 (70.8)	60 (46.2)	84 (67.2)	82 (83.7)	106 (91.4)	**< 0.001**
CHC	137 (29.2)	70 (53.8)	41 (32.8)	16 (16.3)	10 (8.6)	
IDS						
Yes	362 (77.2)	105 (81.4)	96 (77.4)	71 (74.0)	90 (82.8)	0.62
No	103 (22.8)	24 (18.6)	28 (22.6)	25 (26.0)	26 (17.2)	
Medstaff	1.4 (12.3, 2.1)	0.9 (10.3, 1.6)	1.5 (9.3, 2.2)	1.4 (16.8, 2)	1.7 (18.7, 2.2)	**< 0.001**
MedStaffHighlevel	0 (0, 0.05)	0 (0, 0.07)	0 (0, 0.05)	0 (0, 0.04)	0.02 (0, 0.05)	**0.004**
Highlevelstaff (%)	0.07 (0.04, 0.11)	0.09 (0.06, 0.13)	0.07 (0.04, 0.15)	0.05 (0.03, 0.09)	0.06 (0.03, 0.11)	0.16
PBS	39.5 (30.0, 50.0)	40.0 (30.0, 50.0)	39.0 (30.0, 50.0)	30.0 (25.0, 50.0)	35.0 (30.0, 60.0)	0.12

For continuous data, median (P25, P75) was reported, expect for proportion 2016 were reported in mean (standard deviation); Kruskal-Wallis equality-of-populations rank test was used for abnormal variables. The categorical variables were reported in count (column %). There were 4 missing values for IDS. THC, township healthcare center; CHC, community healthcare center; IDS, integrated delivery system; PBS, performance-based salary

### Association between the PCDGS and service scope of primary care facilities

**Fig. 1:**
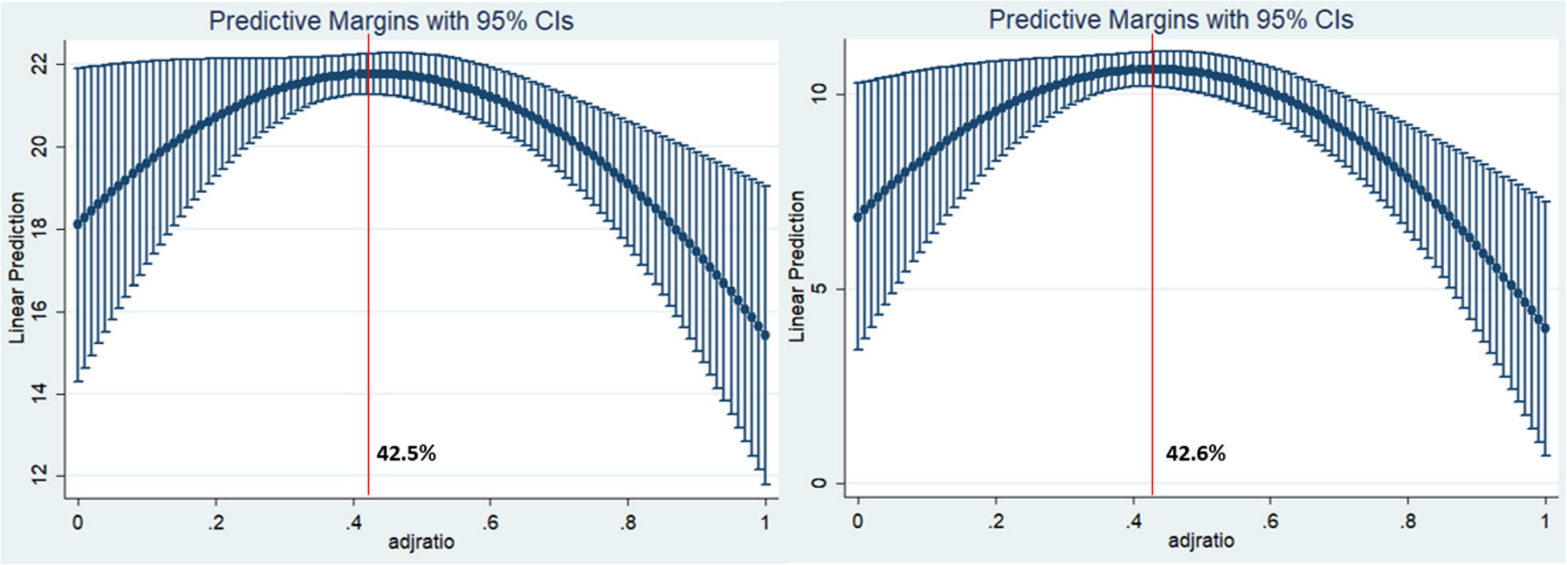
Predictive Margins of PCDGS to service scope in 2017. The solid line plots the Margins with 95% confidence internals were reported. The left panel plots the relationship between PCDGS and service scope of primary care facilities. The right panel plots the relationship between PCDGS and basic medical care service scope of primary care facilities. The red line represents the extremum points (42.5% for left panel and 42.6% for the right panel). CIs, Confidence Intervals; DGS, direct government subsidies; FR, financial revenue

As shown in Table 3, the ordinary least squares model was used to estimate the association between the PCDGS and the service scope of PCFs. We used Poisson and ordinary least squares regression models to explore the association between the PCDGS and the service scope of preventive and public health and basic medical care, respectively. These variables explain approximately 44.5% of the variance in service scope and 39.8% of the variance in the dimension of basic medical care. In Model 1, the coefficient for the linear term of PCDGS is positive and significant (β = 16.52, *P* = 0.034), and the coefficient for the squared term of PCDGS is negative and significant (β = − 19.44, *P* = 0.009), supporting the hypothesis. In Model 2, for the preventive and public health dimension, the coefficient for the linear term of the PCDGS is negative and insignificant (β = − 0.07, *P* = 0.938), and the coefficient for the squared term of the PCDGS is positive and insignificant (β = 0.08, *P* = 0.926). In Model 3, we find that both the coefficients of the PCDGS (β = 17.32, *P* = 0.014) and the squared term of PCDGS (β = − 20.33, *P* = 0.002) are statistically significant. The results of the Sasabuchi-Lind-Mehlum test also supported the hypothesis of an inverted U-shaped relationship with extreme points of 42.5% (*P* = 0.023, 95% CI = 11.59–51.74%) and 42.6% (*P* = 0.010, 95% CI = 22.41, − 50.58%) for the score of service scope and basic medical care dimension, respectively. The marginal effect of the PCDGS is illustrated in Fig. 1. In addition, PCFs located in areas with smaller populations (*P* <  0.001) and in urban areas (*P* <  0.001) had a smaller service scope.

**Table 3:** Results of U-shaped relationship test

Variable	Service scope (Model 1)			Preventive and public health (Model 2)			Basic medical care (Model 3)		
	β	95% CI	***P***	β	95% CI	***P***	β	95% CI	***P***
PCDGS (%)	16.52	(1.28, 31.76)	**0.034**	−0.07	(−1.86, 1.72)	0.938	17.32	(3.56, 31.07)	**0.014**
PCDGS^2^ (%)	−19.44	(−33.85, −5.02)	**0.009**	0.08	(−1.61, 1.77)	0.926	−20.33	(−33.34, −7.32)	**0.002**
PercapitaCFR	0.03	(0, 0.06)	0.074	0.00	(0, 0)	0.892	0.03	(0, 0.06)	**0.03**
PercapitaGDP	−0.10	(−0.25, 0.04)	0.172	0.00	(−0.02, 0.01)	0.792	−0.08	(−0.21, 0.06)	0.259
Pubhospitalave	0.30	(−0.05, 0.66)	0.093	0.01	(−0.03, 0.05)	0.638	0.19	(−0.13, 0.51)	0.244
Prihospitalave	−0.35	(−0.77, 0.07)	0.099	0.00	(−0.05, 0.04)	0.841	−0.29	(−0.67, 0.09)	0.128
Res e	0.04	(0.02, 0.06)	**< 0.001**	0.00	(0, 0)	0.892	0.04	(0.03, 0.05)	**< 0.001**
Type (ref: THC)	−3.75	(−5.03, −2.47)	**< 0.001**	−0.06	(−0.21, 0.09)	0.458	−3.12	(−4.27, −1.96)	**< 0.001**
IDS (ref: Yes)	−0.35	(− 1.44, 0.73)	0.520	0.00	(−0.12, 0.13)	0.969	−0.38	(−1.36, 0.6)	0.441
Medstaff	−0.60	(−1.25, 0.04)	0.067	0.00	(−0.08, 0.08)	0.985	−0.59	(−1.18, − 0.01)	0.046
MedStaffHighlevel	1.70	(−7.32, 10.72)	0.710	0.06	(−0.99, 1.12)	0.908	1.08	(−7.07, 9.22)	0.794
Highlevelstaff (%)	−8.10	(−18.69, 2.49)	0.133	−0.27	(−1.55, 1)	0.676	−5.26	(−14.82, 4.3)	0.279
PBS	0.02	(−0.01, 0.04)	0.179	0.00	(0, 0)	0.986	0.02	(0, 0.04)	0.131
Slope at PCDGS (min)	12.84			\			13.47		
Slope at PCDGS (max)	−20.83			\			−21.75		
*P*	0.022						0.010		
Fieller 95% CI	(11.59%, 51.74%)			\			(22.41%, 50.58%)		

Model 1, F = 11.29, *P* < 0.001, Adj-R^2^ = 44.5%; Model 2, Poisson regression model were used to examine the association between PCDGS and public health dimension score. *P* = 0.986; Model 3, F = 9.50, *P* < 0.001, Ajd-R^2^ = 39.8%; SLM test, Sasabuchi-Lind-Mehlum test

## Discussion

To the best of our knowledge, this is the first study to explore the association between DGS and the service scope of PCFs. The current study extended the existing research by making two new contributions. First, this study measured the service scope of PCFs with two dimensions and compared the scope of primary care services with a range of facility-level and county-level characteristics. This can help enrich the current research on primary care services beyond physician-level activities. Second, our findings revealed that the inappropriate structure of the current financing system for PCFs in China might hinder the service scope of PCFs.

First, the PCDGS of enrolled PCFs was 50.5%, which is higher than the national level of 37% for rural PCFs and 45% for urban PCFs in 2017 [17]. The PCDGS varied largely between the four quartiles of the service scope score. PCFs from communities with more residents provided a broader scope of services. THCs also provided more kinds of services than CHCs did. This indicated that a smaller service scope is more common among PCFs located in townships or communities with smaller populations or PCFs located in urban areas. This is consistent with the findings of one prior study that revealed that rural physicians in Ontario, Canada engaged in a broader scope of clinical activities varied by community need [37]. This is also consistent with the findings of prior studies that limited FR may cause a lack of economies of scale and comprehensiveness of primary care among small, for-profit urban hospitals in highly competitive markets in the United States [11, 12]. Moreover, providers from densely populated areas in the United States often face intensified competition for insured patients [38]. Hospital service closures are mainly concentrated among rural areas in the US [38, 39]. This indicated that PCFs in less populated areas should be strengthened. Moreover, the current study showed that the proportion of medical staff with a high-level technical title was not associated with the service scope of PCFs. This suggests that service scope may be more strongly associated with the work environment beyond human resources. In addition, the current study did not show that the intensity of hospitals could lead to a reduced service scope of PCFs. This is inconsistent with previous findings suggesting that physicians and their associations feel threatened by the potential competition from nurse practitioners and resist expansion [40].

Second, the current results did not support the hypothesis of an inverted U-shaped relationship between the PCDGS and the service scope of preventive and public health. This result is consistent with the findings of one study that found that substantial subsidies reduced the instability of FR and expanded the provision of public health services, especially those facilities from areas with smaller populations or from less-developed areas [41]. One previous study also indicated that physicians who expanded their service scope from medical care services to public health services had to focus on the large quantity of assessments that might reduce the diversity of basic medical care services [16]. Given that preventive and public health services are directly reimbursed by the government and PCFs did not receive compensation for the basic medical services of relatively low prices set by the government [17], some PCFs would like to stop providing high-risk and less-profitable medical services. PCFs would also take these preventive and public health services as priorities due to substantially restricted monitoring. One study on the village health station also revealed that the institutional ambiguity of different level healthcare institutions has negatively affected the performance of the healthcare system [42]. We may cautiously infer that strengthening the primary care system should start with medical services.

Third, with PCDGS reaching the extreme point of approximately 45%, the service scope narrowed, particularly basic medical care services. A possible explanation for the vanishing effect might be related to decreased enthusiasm and subsequent reduced efficiency. This could also be explained by lower job satisfaction among the current medical staff caused by the current salary system [43]. Moreover, the threshold is much lower than the current level of government subsidy depth among the PCFs we studied. This might cause suboptimal allocation of healthcare resources, suggesting financial returns of DGS might be inefficient from the system perspective. This result indicates that inappropriate direct government subsidies might be associated with a high likelihood of specific medical services closing, even though these services are essential and widely covered by the local government. PCFs with larger PCDGS did provide more comprehensive preventive and public health services but paid a price of reduced basic medical care services due to decreased financial revenue by the current pricing regulation and ceiling line set by the medical insurance system [16, 17]. This is consistent with the fact that services provided across the United States Critical Access Hospitals are associated with the portion of charges and cost received from Medicare payment [44]. As the primary care system with comprehensive care demonstrated better health outcomes [45, 46], future reform of the DGS system should establish an evaluation system that incentivizes wider provision of primary care services [47]. Additionally, future policies are warranted to be enacted per local physician’s intention and promote the career advancement of some primary care professionals beyond financial incentives [17, 48]. As autonomy could help better match a PCF’s preferences of service provision to community needs [47], the Chinese government, especially the healthcare security administration, may need to reform programmes of “wrong” subsidies, thereby promoting basic medical care service provision and improving system efficiency. Fortunately, related policies have been enacted to remunerate medical staff with salaries comparable to those of their counterparts from local hospitals [17]. The focus of these policies is to permit PCFs to set their salary level above the ceiling of government departments and to distribute profits made by the service provision for salaries.

### Limitation

This study has several limitations. First, the current study did not take into account the quantity and quality of services provided for the service scope scale, which should be recalibrated in future studies. Second, service scope is based on self-reported data and may be subject to social desirability bias. Facilities included in the current study reported a higher PCDGS compared to the national level, which indicated that the current findings should be generalized cautiously. Third, we could not make the causal inference based on the cross-sectional study. In addition, as a country with a large population and regional diversity, the complex mechanism between the financing structure and primary care service scope remains to be determined by more rigorous studies that go beyond association analyses.

## Conclusion

The disproportionate proportion of direct government subsidies to the financial revenue of PCFs might narrow the service scope of the primary care system with a misaligned incentive structure. This may lead to the underutilization of primary care services and poor performance of the healthcare delivery system. Our findings suggest that future policies are warranted to promote the strategic procurement of primary care services, especially medical services, with a performance-based subsidies system to determine how to allocate financial resources.

## Data Availability

All the research data is available from the correspondence author upon reasonable request.

## Appendix 1

**Table 4:** Six provinces-level regions selected from mainland China

Geographic Location	provinces/autonomous regions/municipalities	Amounts	Selected	economic development level
EasternChina	Liaoning, Beijing, Tianjing, Shanghai,Hebei, Shandong, Zhejing, Fujian, Guangdong, Guangxi, Hainan	12	Shandong	less-developed
			Guangdong	developed
CentralChina	Heilongjiang, Jilin, Shanxi, Inner Mongolia, Anhui, Henan, Hubei, Hunan, Jiangxi	9	Henan	less-developed
			Hubei	developed
WesternChina	Chongqing, Sichuang, Yunan, Guizhou, Tibet, Shaanxi, Gansu, Qinghai, Ningxia, Xinjiang	10	Guizhou	less-developed
			Chongqing	developed

## Appendix 2

**Table 5:** 36 counties/district selected from six provinces-level regions

provinces-level regions	Cities	counties/districts	Urban/Rural
Shandong	Jining	Rencheng	Urban
	Jining	Wencheng	Rural
	Jining	Wenshang	Rural
	Qingdao	Shibei	Urban
	Qingdao	Jiaozhou	Rural
	Qingdao	Laixi	Rural
Guangdong	Shenzhen	Baoan	Urban
	Shenzhen	Futian	Urban
	Shaoguan	Nanxiong	Rural
	Shaoguan	Wenyuan	Rural
	Shaoguan	Ruyuan	Rural
	Shaoguan	Shixing	Rural
Hubei	Yichang	Xiling	Urban
	YIchang	Dangyang	Rural
	YIchang	Xingshan	Rural
	Huanggang	Huangzhou	Urban
	Huanggang	Macheng	Rural
	Huanggang	Xishui	Rural
Henan	Luoyang	Xigong	Urban
	Luoyang	Xinan	Rural
	Luoyang	Luoning	Rural
	Shangqiu	Liangyuan	Urban
	Shangqiu	Liangyuan	Rural
	Shangqiu	Yucheng	Rural
Chongqing		Jiulongpo	Urban
		Yubei	Urban
		Fengjie	Rural
		Zhong	Rural
		Chengzhou	Rural
		Fengdou	Rural
Guizhou	Zunyi	Hongguangang	Urban
	Zunyi	Meitan	Rural
	Zunyi	Yuqing	Rural
	Tongren	Bijiang	Urban
	Tongren	Jiangkou	Rural
	Tongren	Sinan	Rural

## Abbreviations

ED: Emergency department
PCF: Primary care facilities
PBS: Performance-based salary
DGS: Direct government subsidies
FR: Financial revenue
PCDGS: Proportion of cumulative direct government subsidies to financial revenue
THCs: Township healthcare centres
CHCs: Community healthcare centres
